# Current Controversies in the Prediction, Diagnosis, and Management of Cerebral Vasospasm: Where Do We Stand?

**DOI:** 10.1155/2013/373458

**Published:** 2013-10-08

**Authors:** Young Lee, Scott L. Zuckerman, J. Mocco

**Affiliations:** ^1^Vanderbilt University School of Medicine, 1211 Medical Center Drive, Nashville, TN 37232, USA; ^2^Department of Neurological Surgery, Vanderbilt University School of Medicine, 1211 Medical Center Drive, Nashville, TN 37232, USA

## Abstract

Aneurysmal subarachnoid hemorrhage occurs in approximately 30,000 persons in the United States each year. Around 30 percent of patients with aneurysmal subarachnoid hemorrhage suffer from cerebral ischemia and infarction due to cerebral vasospasm, a leading cause of treatable death and disability following aneurysmal subarachnoid hemorrhage. Methods used to predict, diagnose, and manage vasospasm are the topic of recent active research. This paper utilizes a comprehensive review of the recent literature to address controversies surrounding these topics. Evidence regarding the effect of age, smoking, and cocaine use on the incidence and outcome of vasospasm is reviewed. The abilities of different computed tomography grading schemes to predict vasospasm in the aftermath of subarachnoid hemorrhage are presented. Additionally, the utility of different diagnostic methods for the detection and visualization of vasospasm, including transcranial Doppler ultrasonography, CT angiography, digital subtraction angiography, and CT perfusion imaging is discussed. Finally, the recent literature regarding interventions for the prophylaxis and treatment of vasospasm, including hyperdynamic therapy, albumin, calcium channel agonists, statins, magnesium sulfate, and endothelin antagonists is summarized. Recent studies regarding each topic were reviewed for consensus recommendations from the literature, which were then presented.

## 1. Introduction

Aneurysmal subarachnoid hemorrhage (aSAH) is a relatively rare cause of stroke, occurring in approximately 30,000 persons in the United States each year. However, its impact equals that of cerebral ischemia, the most common cause of stroke, due to its higher morbidity, higher mortality, and occurrence in younger individuals. Approximately 20 to 30 percent of patients with aSAH suffer from cerebral ischemia and infarction due to cerebral vasospasm, which is the number one cause of treatable death and disability following aSAH. Vasospasm occurs most frequently at 7 to 8 days after aSAH and can last for a prolonged period. Clinical vasospasm is defined as a decline in neurologic status due to vasospasm and can result in severe morbidity and mortality. However, clinical vasospasm has also been observed after other invasive and traumatic processes such as craniotomy and traumatic brain injury. The pathogenesis and etiology of this vasospasm are very poorly understood, and treatments to prevent post-SAH vasospasm are widely varied and have greatly different magnitudes of effectiveness. New methods to predict, diagnosis, and manage vasospasm are an active field of research, and there is great controversy regarding the effectiveness of these various methods to affect the course of vasospasm. Further research is needed to settle some of these controversies and to establish evidence to justify the use of some of the more common interventions already used in clinical practice. The aim of this paper is to address controversies in the prediction, diagnosis, and management of cerebral vasospasm based on a comprehensive review of the recent literature regarding these topics.

## 2. Outcomes Used in Vasospasm Research

Previous studies on aSAH have studied a variety of different outcomes, making the direct comparison of the results difficult. While delayed cerebral ischemia (DCI) has generally been utilized as the outcome in many studies, its definition has not been applied consistently. While it is defined most accurately as “the occurrence of focal neurological impairment or a decrease of at least 2 points on the Glasgow Coma Scale” which lasts for at least one hour, it is not apparent immediately after aneurysm occlusion, and cannot be due to other causes by means of clinical assessment, CT, MRI, or appropriate laboratory studies. A recent literature review by Vergouwen et al. found that a variety of different terms had been used to reflect DCI, including delayed ischemic neurologic deficit (DIND), secondary cerebral ischemia, symptomatic ischemia, vasospasm, clinical vasospasm, symptomatic vasospasm, and cerebral infarction. While DCI is most purely a clinical definition, many previous studies had combined its definition with angiographic or anatomic evidence of vasospasm, making it impossible to compare many studies. Vasospasm, although often associated with DCI, is defined as an anatomic narrowing of the intracranial arteries from various causes such as constriction, swelling, endothelial remodeling, and fibrosis. According to Poiseuille's law, the resistance of an artery depends on the fourth power of radius. It is hypothesized that this increase in resistance in vasospasm decreases blood flow to dependent regions of the brain, resulting in DCI and eventually cerebral infarction. Diagnosis of vasospasm is also made using a variety of technologies. Vasospasm can be visualized directly with imaging such as angiography or inferred indirectly from increased blood flow velocities as would be expected according to Bernoulli's law in an artery with a decreased luminal cross-sectional area. While it is generally true that vasospasm is strongly associated with DCI [1] and cerebral infarction [2–4], patients with aSAH can have poor clinical and functional outcomes without vasospasm. Likewise, vasospasm can occur in the absence of poor clinical outcomes. At best, vasospasm serves as a surrogate marker for DCI, but it has incorrectly been used as a synonym for DCI in many studies. Recently, the International Multidisciplinary Consensus Conference on the Critical Care Management of Subarachnoid Hemorrhage published guidelines for a uniform definition of DCI for use in clinical trials and observational studies [5]. It recommended that separately defined outcomes for angiographic vasospasm, DCI, cerebral infarction, and functional outcome be utilized in future studies. There was consensus that cerebral infarction on neuroimaging would be a better outcome measure than DCI or vasospasm because it is more objective (with a higher interobserver reliability), has a higher sensitivity in sedated/comatose patients, and has been shown to be a superior predictor of 3-month functional outcome. It is important to interpret the results of the studies described in this paper in this context.

### 2.1. Prediction of Vasospasm

#### 2.1.1. Age

Epidemiological studies indicate that the incidence of aSAH gradually increases with increasing age [6]. There is disagreement in the literature, however, as to the relationship between age and incidence of vasospasm after ruptured aSAH. Several studies have indicated that younger age increases risk of vasospasm [7–12]. Other studies have indicated that older patients are at greater risk of vasospasm [13] or that there is no relationship between age and vasospasm [14–17]. The definition of vasospasm used in these studies differed along with the method of diagnosing vasospasm, which makes the interpretation of these studies difficult. Two of the studies performed only bivariate analyses of age as a predictor of vasospasm and were not controlled for other common confounders [7, 12]. Three studies suggesting an inverse relationship between increasing age and increased risk of vasospasm utilized threshold cerebral blood flow velocity (cBFV) values from transcranial Doppler (TCD) to define vasospasm. The sensitivity of a high cBFV for detecting symptomatic vasospasm in elderly SAH patients has been shown to be particularly low when using the same criteria as in younger patients, because older patients have significantly lower maximum mean cBFVs [18]. In elderly patients, DCI occurred at a lower cBFV [12]. The differences in the definition of vasospasm, measurement modality, and age categories used in these studies make the interpretation of their results difficult. Further research into the effects of age on vasospasm and cerebral infarction is needed.

#### 2.1.2. Smoking

Smoking has been suggested for decades as one of the most important risk factors for subarachnoid hemorrhage and recent evidence firmly supports this relationship [19–21]. Cigarette smoking also appears to increase the risk of angiographic vasospasm and DCI in patients with ruptured aSAH. Two prospective studies found that cigarette smoking was an independent predictor of DCI after aSAH [15, 22]. This is plausible, as cigarette smoking has previously been identified as the most important risk factor in the development of coronary arterial vasospasm [23–25]. However, it is unclear which components of cigarette smoke are associated with the increased risk of symptomatic vasospasm. A retrospective study involving 258 active smokers found that there was a decreased risk of symptomatic vasospasm amongst those given nicotine replacement therapy. Yet another recent retrospective study of a similar size showed no association between nicotine replacement therapy and vasospasm or DCI [26]. There is yet no consensus on the effects of nicotine on cerebral vasculature. However, there is consensus in the recent literature suggesting that smoking is associated with an increased risk of DCI after aSAH.

#### 2.1.3. Cocaine Use

Cocaine use can also cause coronary artery vasospasm and is one established cause of Prinzmetal angina. Additionally, it has been associated with neurological complications such as intraparenchymal hemorrhage, intraventricular hemorrhage, and cerebral infarction. However, there have been fewer studies on the angiographic effects of cocaine on human cerebral arteries. There is controversy in the literature on the effect of cocaine use in the aftermath of aSAH. In 2001, Conway and Tamargo determined that of 440 patients who experienced aSAH over a 7-year period, 27 patients had used cocaine within 72 hours of aSAH rupture. Cocaine use was found to be an independent predictor of DCI with an odds ratio of 6.41 (95% CI, 2.14–19.24, *P* = 0.0009) [27]. More recently, Boco and Macdonald reported no significant differences in mean internal carotid, middle cerebral, anterior cerebral, and basilar artery angiographic diameters between a group of 13 cocaine users and 26 control aSAH patients [28]. A study in 2010 casts further doubt on the association between cocaine use and vasospasm. Alaraj et al. showed that of 600 patients with aSAH included in the study, 31 (5%) had a history of recent cocaine use [29]. Cocaine users were younger (45.1 years versus 54.1, *P* < 0.0003) and were more likely to also use tobacco and alcohol. However, no association was found between cocaine use and Hunt-Hess score, Fisher grade, short-term outcome (modified Rankin scale >3), symptomatic vasospasm, radiologic vasospasm, stroke, or mortality. The largest study to date of 142 patients with cocaine use-associated aSAH by Chang et al. in 2013 reported that cocaine users tended to be younger (49 years versus 53 years, *P* < 0.001) and had no difference in Hunt-Hess score, intraventricular hemorrhage, and hydrocephalus, which was consistent with previous studies. Most importantly, however, cocaine use was not an independent predictor of DCI when controlled for with other known independent predictors such as age, Hunt-Hess, WFNS, and admission Glasgow Coma Scale (GCS) score. This result conflicts with the study by Conway et al. but had a much larger sample size (*n* = 142 versus *n* = 27 cocaine users). While cocaine users were more likely to have hospital mortality with an odds ratio of 2.9 (95% CI, 1.76–4.63, *P* < 0.001), there was no difference in 3-month functional outcome. Overall, there seem to be few studies on the effect of cocaine use on vasospasm after aSAH and there is no definite consensus between them. There is a need for well-powered studies with well-defined outcomes comparing angiographic vasospasm, clinical outcome, cerebral infarction, and mortality in cocaine-associated aSAH.

### 2.2. Computed Tomography Imaging

The relationship between the amount and density of blood detected on an initial computed tomography (CT) scan of patients admitted for a diagnosis of ruptured aSAH and angiographic vasospasm was first observed in the late 1970s. In 1980, Mizukami et al. found that amongst 26 patients with high density hemorrhage on initial CT scans, 84.6% developed cerebral vasospasm while none of 8 patients did not develop cerebral vasospasm [30].

#### 2.2.1. Timing

It is universally accepted that early CT is the cornerstone of SAH diagnosis. Additionally, earlier CT scans have greater predictive power for symptomatic vasospasm. In 1980, Davis et al. demonstrated a direct correlation between the extent of blood and the severity of vasospasm that developed only in CT scans taken within 4 days of SAH [31]. More recently, Dupont et al. found that there was a relationship between the extent of subarachnoid blood on admission CT imaging and the subsequent development of vasospasm only if the CT was performed within 24 hours of aneurysmal rupture [32]. CT scans performed outside this window showed no correlation with symptomatic vasospasm. These data suggest that the earlier the CT scan after ruptured aSAH, the greater the prognostic value in predicting cerebral vasospasm.

#### 2.2.2. Location and Amount of Blood

One of the best predictors of cerebral vasospasm to date is the characteristic of aSAH on admission CT shortly after aneurysmal rupture of the aneurysm [33, 34]. The most commonly used and well-known grading scale for classification of admission CT is the Fisher grading scale [35, 36]. In the well-known analysis between the amount of subarachnoid blood and development of cerebral vasospasm, Fisher et al. found that there was a high correlation between symptomatic vasospasm and Grade III SAH, classified by the presence of subarachnoid clots greater than 5 × 3 mm in diameter in cisterns and fissures in the horizontal plane, or clots greater than 1 mm thick in the vertical plane [35, 36]. In 2001, Classen et al. suggested an aSAH grading system based on an analysis of 276 patients who had a CT scan within 72 hours of rupture, which found that the best predictors of DCI were blood in both lateral ventricles (odds ratio (OR) 4.1, 95% CI 1.7–9.8) and a thick clot completely filling any cistern or fissure (odds ratio (OR) 2.3, 95% CI 1.5–9.5). These two characteristics were found to be independently predictive of cerebral vasospasm, and the Claassen scale incorporated separate categories for cisternal blood with and without bilateral intraventricular hemorrhage. 

The role of cisternal blood in predicting vasospasm has been firmly established in the literature. In 1978, Takemae et al. reported that there was a significant relationship between cerebral vasospasm and a high density of blood in basal subarachnoid cisterns on admission CT scans. In a report of the cooperative aneurysm study, Adams et al. discovered that focal, thick collections of blood in the cisternae were highly predictive of DCI [37]. Grosset et al. studied the admission CT scans of 121 patients presenting after aSAH [38]. The presence of intracisternal blood on admission CT predicted DCI. 

The role of intraventricular blood in predicting cerebral vasospasm is less clear. There have been published studies indicating no relationship between IVH on admission CT scan and symptomatic vasospasm [31, 37, 38]. The majority of studies, however, found that IVH was predictive of symptomatic vasospasm [31, 34, 39–42]. A modified Fisher CT grading scale taking into account thick cisternal and ventricular blood outperformed the original Fisher scale in predicting symptomatic vasospasm in a study of 1355 aSAH patients in the placebo arm of a randomized controlled trial (RCT) of tirilazad [43]. In 2011, Ko et al. used imaging software to calculate cisternal plus intraventricular hemorrhage volume (CHIV) from CT scans taken within 24 hours of aneurysmal rupture. Patients in the highest quartile of CHIV (≥31.0 mL) were significantly more likely to develop DCI (odds ratio (OR) 4.6, 95% CI 1.34–27.83) than those in the lowest quartile (<9.6 mL) [44]. It seems clear that greater amounts of blood predict greater risk of symptomatic vasospasm. Cisternal blood volumes have uniformly been found to be a predictor of symptomatic vasospasm, and recent research utilizing large datasets indicates that intraventricular hemorrhage is also predictive, although this has been controversial.

#### 2.2.3. Grading Scheme

The Fisher scale is currently the most widely used grading scheme for CT scan. Advocates emphasize its ease of use and predictive power [45]. However, other authors have voiced concerns over several limitations in the implementation and effectiveness of the scheme. The highest score (Grade IV SAH) does not have the highest risk of vasospasm, which can be confusing, and there is no category for patients who have both thick cisternal blood and intraventricular hemorrhage, even though these may be independent and additive predictors of vasospasm [40]. In a randomized controlled trial of nimodipine in patients with SAH, investigators had difficulty in differentiating between Grade II and Grade III SAH [46]. Smith et al. found that in modern practice, the Fisher grade only predicted symptomatic vasospasm in half of the patients [42]. Van Norden et al. also noted that Fisher Grade III and Grade IV did not predict DCI [47]. This may indicate that in the era of modern practice, where nimodipine, hypertensive therapy, and endovascular therapy have become established, the Fisher grade may be outdated. 

In addition, many studies have raised concern that the Fisher scale is a qualitative, categorical scale and may have considerable interobserver variability that may adversely affect its usefulness for clinical practice and research. An earlier study by Ogilvy and Carter collapsed the Fisher scale into Grades 0 to II versus Grades III and IV and noted that there was almost perfect agreement (kappa 0.90) [48]. However, the utility of such a collapsed grading scale is questionable. Ibrahim et al. observed that the Fisher scale had only fair-to-moderate agreement (kappa 0.41, 95% CI 0.33–0.49) compared to a semiquantitative scale, the Hijdra scale (ICC 0.56, 95% 0.49–0.62) [49]. Van Norden et al. also found the Hijdra scale (kappa ranging from 0.67 to 0.75) to have greater interobserver reliability than the Fisher scale (kappa ranging from 0.37 to 0.55). Svensson et al. found slightly greater agreement for the Fisher scale (kappa 0.50–0.63, moderate to substantial agreement) [50]. Kramer et al. published consistent results observing that the Claassen scale had substantial agreement (kappa 0.64), while the modified Fisher scale and Fisher scale had moderate agreement (kappa 0.59 and 0.45, resp.). The modified Fisher scale, however, was the only grading scale in which each unit increase was associated with an incremental risk for all 3 outcomes and was thus recommended by the authors [51]. The Hijdra scale was also found to be superior to the original Fisher scale in terms of interobserver reliability and ability to predict symptomatic vasospasm [47, 49, 52]. It has been criticized by other authors, however, for being difficult to use in clinical practice [53]. While there are a variety of grading schemes for interpretation of CT scans, each possesses limitations to its validity, both internal and external, and/or ease of implementation in the clinical practice setting. None of these grading schemes are accepted universally [54]. Future research is needed to produce a valid, reliable grading scheme that provides strong prognostic information for development of symptomatic vasospasm.

### 2.3. Diagnostic Methods for Vasospasm

Various methods are currently used to detect vasospasm including TCD, CT angiography, digital subtraction angiography (DSA), and CT perfusion imaging. Of these, TCD is the most widely used but the standard of reference for the anatomic demonstration of cerebral vasospasm is DSA. However, DSA is costly and has a small but nonnegligible risk of neurologic complications, making the consideration of the other modalities of diagnosis attractive [55]. However, there is controversy over which method is the best at detecting vasospasm. While TCD is inexpensive, available at the bedside, noninvasive, and without known adverse side effects, it is operator dependent, requires a good acoustic window, and has been known to have a high false negative rate for vasospasm when compared to DSA [56, 57]. It has been shown to be specific, but not sensitive for vasospasm and poorly predictive of developing secondary cerebral infarction compared to angiography [58, 59]. CT perfusion imaging has shown good specificity and accuracy in detecting vasospasm with a specificity of 100% and an accuracy of 92.3% [60]. Specifically, the cerebral blood flow reduction on perfusion CT was found to significantly predict the clinical outcome of patients. A meta-analysis comparing the accuracy of CT angiography and CT perfusion for detecting vasospasm found that they had similar specificity and sensitivity, with an overall sensitivity and specificity of 79.6% and 93.1%, respectively, for CT angiography and 74.1% and 93.0%, respectively, for CT perfusion [61]. Unfortunately, both of these modalities can be limited by beam-hardening artifact from clips and coils and are of limited utility in evaluating posterior fossa territories. Because of the different detection characteristics for these modalities, there is controversy over the modern incidence of vasospasm and that with increased specificity of more recent techniques, the actual incidence of vasospasm may be lower than noted in previous studies. A prospective study systemically comparing the ability of these techniques to predict DCI would provide valuable evidence to guide monitoring strategies for vasospasm.

### 2.4. Hyperdynamic Therapy

A mainstay in the prophylaxis and treatment of cerebral vasospasm in the past was hyperdynamic therapy, also known as “triple-H therapy,” which utilized the three approaches of hypervolemia, induced hypertension, and slight hemodilution with the aim of improving cerebral blood flow (CBF). In contemporary practice, there is much disagreement on the use of hyperdynamic therapy in the prophylaxis and treatment of vasospasm after aSAH. A survey of 626 physicians (anesthesiologists, internists, neurologists, and neurosurgeons) in Europe and the United States found that 39% and 52% of respondents would use hyperdynamic therapy for the prophylaxis and treatment of vasospasm after aSAH, respectively, [62]. These approaches are usually attempted after the repair of the aneurysm. This combined treatment approach has not yet been assessed for efficacy in any large RCTs, and as such, evidence for efficacy has been based mostly on case series of patients with DCI attributed to vasospasm. Although originally patients were treated with a combination of all three of these therapies (hypervolemia, hypertension, and hemodilution), recently the efficacy of each component of “triple-H” therapy has been questioned. In addition, studies have been conducted recently to specifically address the role of these therapies to prophylactically prevent vasospasm.

#### 2.4.1. Hyperdynamic Therapy as Prophylaxis

In the early 2000s, two RCTs compared the effect of prophylactic hypervolemia to normovolemia. In 82 patients assigned to prophylactic normovolemia or hypervolemia after aneurysm clipping, Lennihan et al. demonstrated that hypervolemic therapy resulted in increased cardiac filling pressures and fluid intake but did not increase CBF or blood volume compared with normovolemic therapy, concluding that prophylactic hypervolemic therapy is unlikely to confer benefit [63]. In 2001, Egge et al. published results from an RCT of 32 patients who were randomized to prophylactic normovolemia or “triple-H” therapy. They observed no differences between the two groups with respect to vasospasm or DCI [64]. In addition to having no benefit, prophylactic hyperdynamic therapy was found to have greater costs and more frequent complications such as excess bleeding, congestive heart failure, and infections [64]. A recent, comprehensive review of 11 studies in the literature on prophylactic hyperdynamic therapy concluded that the available evidence failed to show a benefit for the use of prophylactic hyperdynamic therapy and suggested harm in using overly aggressive hydration [65]. Based on current published data, routine prophylactic hyperdynamic therapy cannot be recommended.

#### 2.4.2. Hyperdynamic Therapy as Treatment

There seems to be evidence that suggests that the use of hemodynamic therapy after the occurrence of vasospasm may be beneficial and could reverse symptomatic vasospasm and neurological deficits. In 1982, Kassell et al. induced arterial hypertension in 58 patients with intravascular volume expansion, blockade of the vagal depressor response, and the administration of antidiuretics and vasopressor agents, resulting in a permanent reversal of neurological deterioration in 43 patients [66]. In 1995, Mori et al. found that hypervolemic hemodilution therapy after symptomatic vasospasm could reverse neurological deterioration due to cerebral vasospasm [67]. In 2003, a systematic review of 4 prospective studies of “triple-H” therapy found that it reduced symptomatic vasospasm (relative risk (RR) 0.45, 95% CI 0.53–0.87) and death (RR 0.68, 95% CI 0.53–0.87) but had no effect on DIND (RR 0.54, 95% CI 0.2–1.49) [68]. However, the individual roles of the separate components of “triple-H” therapy are still controversial. 

#### 2.4.3. Induced Hypertension

A study of the recent literature indicates that hypertension may be the most important component of hyperdynamic therapy. A retrospective study of 45 patients conducted in 2005 by Raabe et al. found that hypervolemia could be associated with increased risks but that moderate hypertension (cerebral perfusion pressure 80–120 mmHg) in a normovolemic, hemodiluted patient helped to improve cerebral oxygenation and had a lower complication rate compared to hypervolemic therapy [69]. More recently in 2010, Frontera et al. found that 43% of patients having volume expansion after symptomatic vasospasm had a clinical improvement, while 68% of those with hypertensive therapy responded, suggesting that induced hypertension was more effective in treating vasospasm. In 1990, Otsubo published a study reporting that induced hypertension in the presence of normovolemia reduced the signs and symptoms of vasospasm in 54% of patients [70]. A possible drawback to the use of hypertensive therapy is the possibility of rebleeding, rerupture, or rupture of other, unsecured aneurysms. However, recent evidence seems to suggest that hypertensive therapy is generally safe for secured, ruptured aneurysms and unruptured aneurysms. In 2002, Hoh et al. observed that amongst 40 patients requiring blood pressure augmentation up to 200 mmHg with unsecured unruptured aneurysms, no bleeding events occurred [71]. In a study of 25 consecutive patients with aSAH undergoing induced hypertension after coiling up to a peak systolic blood pressure from 195 mmHg to 205 mmHg, there were no episodes of aneurysm rebleeding [72]. In 2011, Platz et al. conducted a retrospective study of 71 unsecured, unruptured aneurysms with an average size of 4.0 mm during induced hypertension and found that no aneurysm ruptured during therapy [73]. These studies suggest that induced hypertension is efficacious independent of hypervolemia and that it should not be omitted based on the presence of unsecured aneurysms. With evidence cautiously suggesting the efficacy of hypertensive therapy in preventing DCI with a low risk of aneurysmal rupture, it seems reasonable to recommend it for patients with symptomatic vasospasm. Further research is needed in the form of a large RCT, however, to elucidate the role of induced hypertension and to determine its risk profile in patients with symptomatic vasospasm.

#### 2.4.4. Hypervolemia

In patients undergoing surgery for acute aSAH, there is usually a decrease in circulating blood volume for at least 3 days after surgery. Therefore, there is at least theoretical basis in the reasoning that the maintenance of normovolemia via relative hypervolemia within 3 days after surgery for aSAH may improve outcomes. However, hypervolemia has been associated with a higher rate of complications such as pulmonary edema and congestive heart failure [69]. In 2002, Ekelund et al. published a study comparing the effects of iso- and hypervolemic hemodilution on regional cerebral blood flow and oxygen delivery in patients with TCD-defined vasospasm [74]. No difference was found between normovolemic and hypervolemic hemodilution, and rather hemodilution was found to result in a pronounced reduction in oxygen delivery capacity. However, in 2005, Jost et al. reported that the administration of a saline bolus of 15 mL/kg/1 hr in patients with normovolemia at baseline with new symptoms of vasospasm produced an increase in CBF [75]. In 2007, Tseng et al. reported that in patients with a poor-grade SAH, administration of 2 mL/kg of 23.5% hypertonic saline solution decreased intracranial pressure and increased cerebral perfusion pressure, transcranial Doppler velocities, and CBF [76, 77]. The literature regarding hypervolemic therapy is therefore still inconclusive regarding the efficacy of hypervolemia. Despite this, there is some evidence that hypovolemia is associated with increased risk of DCI [78], and therefore, normovolemia should be attained via initial fluid resuscitation in patients with hypovolemia.

#### 2.4.5. Hemodilution versus Transfusion

Hemodilution, particularly the lowering of hematocrit, attempts to decrease the viscosity of blood and thereby improves blood flow to ischemic regions of the brain via the Poiseuille equation, which states that the resistance to flow is inversely proportional to viscosity. However, hemodilution also decreases the oxygen carrying capacity of the blood. An optimal hematocrit aims to increase oxygen delivery to ischemic tissues by balancing these two opposing factors. Based on studies performed in canines, Tummala et al. recommended maintaining a hematocrit of 30–32% utilizing hemodilution with colloids to maximize oxygen delivery to ischemic brain in the setting of vasospasm [79]. In humans, the role of hemodilution in treatment of cerebral vasospasm has also been scrutinized, but with no good consensus in the literature. In 2006, a study by Naidech et al. found that aSAH patients with higher initial and mean hemoglobin values had improved outcomes [80]. A larger study in 2007 of 611 consecutive patients by the same authors supported their earlier conclusions. Higher hemoglobin levels were associated with improved outcomes at 14 days and 3 months, but the authors did not recommend an optimal goal hemoglobin nor routine packed red blood cell transfusions [81]. However, in 2004, Smith et al. retrospectively reviewed the management of 441 patients and found that postoperative packed RBC transfusion actually had a positive correlation with angiographic vasospasm with an odds ratio of 1.68 (95% CI, 1.02–2.75) Additionally, intraoperative RBC transfusion was found to be correlated with worse outcome. The authors concluded that patients should be assessed for anemia to determine whether such transfusion would be necessary [82]. A recent 2010 study by Levine et al. suggested that RBC transfusions are associated with medical complications such as infection. The authors cautiously declared that the data did not infer causation and that further study was necessary to better define the indications for transfusion after SAH [83]. An RCT of 44 patients conducted by Naidech et al. and published in 2010 found no significant difference in cerebral infarction between a hemoglobin goal concentration of at least 10 or 11.5 g/ld. but a trend favoring higher goal hemoglobin (*P* > 0.1) [84]. This suggested that perhaps higher goal hemoglobin in aSAH patients may be feasible and that a larger RCT to determine optimal goal hemoglobin may be warranted. Contrary results were obtained in two separate computational studies modeling the fluid dynamics of blood flow in the middle cerebral artery which showed that hemodilution did not improve oxygen transport, but to the contrary, ischemia may be worsened [85, 86]. These same models also showed that the middle cerebral artery required a large increase in intracranial artery inlet pressure for a beneficial effect on CBF, with a minimal effect of decreased hematocrit. In 2012, neither routine hemodilution nor transfusion was recommended for routine treatment of DCI or vasospasm in the Guidelines for the Management of Aneurysmal Subarachnoid Hemorrhage by the American Stroke Association and based on the current literature; this seems reasonable [87].

#### 2.4.6. Hemodilution with Albumin

There were conflicting results in the literature regarding the usage of albumin for intravascular volume expansion. In 1987, Yamakami et al. administered 5% albumin (500 mL in 30 minutes) to assess the effect of volume expansion and found a reduction in CBF following albumin administration in patients with vasospasm during the first 2 weeks after aSAH with no net change in the 3rd week. Patients without vasospasm did not experience any change in CBF [88]. More recently, in 2004, Suarez et al. found that there was a beneficial effect of albumin when used as a fluid for volume expansion in patients with SAH. Patients in the albumin group were more likely to have better outcomes at 3 months [89]. More recent studies are needed to readdress this topic and no conclusion on albumin therapy can be drawn at this point.

### 2.5. Calcium Channel Antagonists

The first randomized controlled trial of nimodipine in patients with subarachnoid hemorrhage was published by Allen et al. in 1983 in the New England Journal of Medicine [46]. The placebo group had neurologic deficit that was severe or resulted in death by the end of the 21-day treatment period in 8 of 60 patients, while the nimodipine treatment group had an occurrence of 1 of 56 (*P* = 0.03). No side effects from nimodipine were reported. At the time, the authors presumed that the efficacy of nimodipine resulted from its effects on the prevention of cerebral arterial spasm. In 1998, however, a systematic review by Feigin et al. found that nimodipine reduced the frequency of ischemic deficit by 33% (95% CI, 25%–41%) but it has no significant effect on angiographically detected cerebral vasospasm [90]. Thus, it is now thought that the beneficial effect of nimodipine may be through neuroprotective factors rather than the prevention of vasospasm. The use of nimodipine in the prevention of symptomatic vasospasm is no longer a controversial topic in cerebral vasospasm management. There is consensus in the published literature supporting the safety of nimodipine and its efficacy to improve clinical outcomes in patients with aSAH. At least 5 double-blind placebo-controlled trials have consistently demonstrated that nimodipine is efficacious in improving morbidity and mortality in patient with aSAH and is safe for routine use with mild hypertension occurring as a relatively infrequent adverse event [46, 91–94]. An extensive and exhaustive systematic review by The Cochrane Collaboration of RCT of oral nimodipine showed that prophylactic oral nimodipine reduced the risk of poor outcome defined as death or dependence (risk ratio (RR) 0.67, 95% CI 0.55–0.81, sample size 853) and secondary ischemia (risk ratio (RR) 0.64, 95% CI 0.49–0.83, sample size 390) [95]. Nimodipine is now accepted as efficacious, safe, and cost-effective and is recommended as standard of care [87, 96–100]. 

### 2.6. Statins

Statins are widely utilized for their cholesterol lowering effect, but they also exhibit many pleiotropic actions [101]. They have been known to improve endothelial function, possibly via direct upregulation of endothelial nitrogen oxide synthase [102, 103]. In 2002, McGirt et al. published the first results that showed the effect of statins on cerebral vasospasm [104]. Recently, there has been controversy over the prophylactic use of statins to prevent the occurrence of cerebral vasospasm. There have been at least 4 RCTs [105–108] and 6 observational studies [104, 109–114] published on the effects of statins of symptomatic vasospasm. A meta-analysis in 2006 by Sillberg et al. based on 3 RCTs and 158 patients found that the incidence of vasospasm (RR = 0.73, 95% CI 0.54–0.99), delayed ischemic deficits (RR = 0.38, 95% CI 0.17–0.83), and mortality (RR = 0.22, 95% CI 0.06–0.82) were reduced in the group receiving statin versus placebo [115]. More mixed results were found by Kramer and Fletcher who in 2009 performed a meta-analysis of the same 3 RCTs and an additional RCT published in 2009 by Vergouwen et al., and 2 “pseudo-RCTs” which were published as abstracts in the literature [116]. They found that amongst a total of 309 patients, statins significantly reduced the occurrence of DINDs (OR 0.38, 95% CI 0.23–0.65) but did not reduce the occurrence of mortality (OR 0.51, 95% CI 0.25–1.02) or poor neurological recovery (OR 0.81, 95% CI 0.49–1.32). In the same year, Vergouwen et al. performed another meta-analysis on just the 4 truly randomized RCTs and found that statin use did not significantly reduce radiographic vasospasm, DCI, poor outcome, or mortality [117]. These inconclusive results highlight the need for future, well-designed RCTs to determine the effectiveness of statins in preventing symptomatic vasospasm. Currently, there are four such controlled RCTs ongoing and results from these studies are expected in the near future.

### 2.7. Magnesium Sulfate

Magnesium is a widely used, a cost-effective therapy that is well-established in the fields of obstetrics and cardiology [118]. Its effectiveness in the prevention of cerebral vasospasm after aSAH, however, is much more controversial. Although the exact neuroprotective mechanism of magnesium remains speculative [119, 120], there exists a sound theoretical framework to suggest that magnesium sulfate administration may be beneficial after aSAH. Magnesium is known to dilate cerebrovascular arteries, block glutamate release and neurotoxicity [121], block the glutamate NMDA receptor [122], voltage-dependent calcium channels, and buffer against the depletion of intracellular ATP. Promisingly, in numerous earlier small pilot clinical trials, magnesium sulfate showed a trend towards improving clinical outcomes in patients with aSAH [123–130]. However, the results of three recent Phase III, randomized, placebo-controlled, double-blind clinical trials of magnesium sulfate have shown that intravenous magnesium sulfate may not be as effective as previously hoped. A study in 2010 by Wong et al. found that in 327 patients randomized to intravenous magnesium sulfate or saline placebo for 10 to 14 days, 64% of the magnesium sulfate group and 63% of the placebo group had a favorable outcome at 6 months using the extended Glasgow Outcome Scale [131]. Secondary outcome analyses such as the modified Rankin scale, Barthel index, SF-36, and clinical vasospasm also showed no significant differences. Consistent with these finding, no differences in radiographic vasospasm defined by changes in TCD mean MCA velocities were found. In a study published in 2010, Westermaier et al. reported that amongst 110 patients randomized to 10 days of intravenous magnesium sulfate to titrate serum magnesium to 2.0–2.5 mmol/L and control, the incidence of delayed ischemic infarction was significantly lower in the magnesium group (OR 0.28, 95% CI 0.12–0.64) than in the control group [132]. However, this did not translate into a difference in the number reaching good outcome (63% in magnesium versus 51% in control, *P* = 0.209) or in DIND (OR 0.51, 95% CI 0.20–1.29). Interestingly, there was a difference in TCD and DSA defined radiographic vasospasm (67% in magnesium versus 85% in control, *P* = 0.028). The results of the latest Phase III clinical trial published in 2012 by Dorhout Mees et al. showed that in 1204 patients randomized to magnesium and placebo, there was no difference in the incidence of poor outcome (RR 1.03, 95% CI 0.85–1.25) [95]. The authors also performed an updated meta-analysis with 2047 patients, the largest study to date, and concluded that magnesium is not superior to placebo for reduction of poor outcome after aSAH and that routine administration of magnesium after aSAH could not be recommended. These results were consistent with another recent meta-analysis by Wong et al. which included a total of 875 patients and found no difference in DCI (RR 0.87, 95% CI 0.36–2.09), delayed cerebral infarction (RR 0.61, 95% CI 0.35–1.22), favorable outcome at 3 months (RR 1.14, 95% CI 0.99–1.31), or favorable outcome at 6 months (RR 1.08, 95% CI 0.94–1.24). These recent reviews incorporating the latest Phase III clinical trial data suggest that magnesium sulfate may not be as beneficial in patient outcomes after aSAH as previously thought. Based on this new evidence, the routine administration of magnesium sulfate after aSAH cannot be recommended.

### 2.8. Endothelin Antagonists

Endothelin is a 21-residue vasoconstrictor peptide which was discovered as one of the most potent vasoconstrictors known in 1998 by Yanagisawa et al. [133]. A specific subtype of endothelin, endothelin-1, drew much attention as a target for prevention of cerebral vasospasm because it was shown to produce dose-dependent and very long-lasting constrictive effects on cerebral vasculature both in vivo and in vitro [133–136]. There are at least three different peptides in the endothelin family. Three endothelin receptor subtypes also exist. The Et_a_ receptor is located in vascular endothelial cells and is known to mediate the endothelium-dependent vasodilative actions of endothelins. Recently, a specific antagonist to the Et_a_ receptor, clazosentan (Actelion Pharmaceuticals, Allschwil, Switzerland), has been the focus of research. An initial phase IIa study, “Clazosentan to Overcome Neurological Ischemia and Infarction Occurring After Subarachnoid Hemorrhage-1 (CONSCIOUS-1),” was performed. In this randomized, placebo-controlled, double-blind study by Macdonald et al., the highest dose of clazosentan, 15 mg/hr, demonstrated a relative risk reduction in angiographic vasospasm of 65% (95% CI 47–78%; *P* < 0.0001). In a phase III trial of aneurysmal clipping patients by the same investigators, CONSCIOUS-2, with 1157 patients, clazosentan had no significant effect on the primary endpoint, which was defined as mortality or cerebral vasospasm related morbidity 6 weeks after SAH. There was also no significant effect on poor functional outcome at week 12, which was defined as a GOS-E score of at least 4 [137]. A similar phase III study, CONSCIOUS-3, was conducted in patients with aSAH secured by endovascular coiling [138]. Patients received up to 14 days of intravenous clazosentan (5 or 15 mg/h) or placebo. Consistent with the results of CONSCIOUS-2, there was no effect of the lower dose of clazosentan, 5 mg/h, on the primary outcome, defined as all-cause mortality, cerebral vasospasm-related new cerebral infarcts, DIND, or rescue therapy. There was also no effect of this lower dose on the secondary outcome, defined as a GOS-E of at least 4 at week 12. However, the larger dose of clazosentan, 15 mg/h, did significantly reduce cerebral vasospasm-related morbidity and mortality within 6 weeks aSAH. The authors theorized that the complications of clazosentan, including pulmonary complications, anemia, and hypotension, may have biased the effect of clazosentan on cerebral vasospasm towards the null. These recent phase III trials showed a promising statistically and clinically significant effect of endothelin-1 receptor antagonist on angiographic vasospasm. However, this did not translate into improved outcomes at 12 weeks after SAH. Further research is needed to determine whether this failure of clazosentan to affect long-term outcomes is due to its associated side effects or due to the lack of a relationship between angiographic vasospasm and outcomes. 

## 3. Conclusion

This review has focused on some risk factors for vasospasm and DCI after aSAH, the use of CT imaging schemes to grade aSAH. Additionally, it presents recent data on methods to prevent and treat vasospasm after aSAH. Experimental strategies that may be useful in the near and distant future are also discussed. Nimodipine specifically remain the only proven method of preventing vasospasm and DCI. Hemodilution and hypervolemia, components of triple-H therapy which remains a mainstay of vasospasm treatment in the modern practice of neurosurgery, have been controversial. Recent evidence cautiously suggests that hypertension may be the crucial factor for vasospasm treatment. Other therapies, such as magnesium sulfates, statins, and endothelin-1a receptor antagonists showed initial promise but have shown disappointing results in the most recent literature and are thus unproven. Interestingly, roughly one-third of patients develop DCI without angiographic vasospasm, which suggests that vasospasm may be only part of the picture in DCI development after aSAH. Recent research efforts have been vigorously undertaken to elucidate the role of other factors such as delayed effects of global cerebral ischemia, thromboembolism, microcirculatory dysfunction, and cortical spreading depression, which likely also play a role in the outcome after aSAH [139]. In this literature review, we have chosen to focus on the factors specifically related to vasospasm. In the future, hypotheses based on the known pathophysiology of vasospasm are expected to lead to well-powered RCTs that will help to elucidate the efficacy of these unproven management strategies for the prevention and treatment of vasospasm after aSAH.

## Abbreviations

aSAH: Aneurysmal subarachnoid hemorrhage
TCD: Transcranial doppler ultrasonography
DCI: Delayed cerebral ischemia
DIND: Delayed ischemic neurologic deficit
CT: Computed tomography.
